# Bionic Silk Fibroin Film Induces Morphological Changes and Differentiation of Tendon Stem/Progenitor Cells

**DOI:** 10.1155/2020/8865841

**Published:** 2020-12-01

**Authors:** Kang Lu, Xiaodie Chen, Hong Tang, Mei Zhou, Gang He, Juan Liu, Xuting Bian, Yupeng Guo, Fan Lai, Mingyu Yang, Zhisong Lu, Kanglai Tang

**Affiliations:** ^1^Department of Orthopedics/Sports Medicine Center, State Key Laboratory of Trauma, Burn and Combined Injury, Southwest Hospital, Army Medical University (Third Military Medical University), Chongqing 400038, China; ^2^Institute for Clean Energy & Advanced Materials, School of Materials & Energy, Southwest University, Chongqing 400715, China

## Abstract

**Purpose:**

Tendon injuries are common musculoskeletal system disorders, but the ability for tendon regeneration is limited. Silk fibroin (SF) film may be suitable for tendon regeneration due to its excellent biocompatibility and physical properties. This study is aimed at evaluating the application value of bionic SF film in tendon regeneration.

**Methods:**

Tendon stem/progenitor cells (TSPCs) were isolated from rat Achilles tendon and characterized based on their surface marker expression and multilineage differentiation potential. SF films with smooth or bionic microstructure surfaces (5, 10, 15, 20 *μ*m) were prepared. The morphology and mechanical properties of natural tendons and SF films were characterized. TSPCs were used as the seed cells, and the cell viability and cell adhesion morphology were analyzed. The tendongenesis-related gene expression of TSPCs was also evaluated using quantitative polymerase chain reaction.

**Results:**

Compared to the native tendon, only the 10, 15, and 20 *μ*m SF film groups had comparable maximum loading and ultimate stress, with the exception of the breaking elongation rate. The 10 *μ*m SF film group had the highest percentage of oriented cells and the most significant changes in cell morphology. The most significant upregulations in the expression of *COL1A1*, *TNC*, *TNMD,* and *SCX* were also observed in the 10 *μ*m SF film group.

**Conclusion:**

SF film with a bionic microstructure can serve as a tissue engineering scaffold and provide biophysical cues for the use of TSPCs to achieve proper cellular adherence arrangement and morphology as well as promote the tenogenic differentiation of TSPCs, making it a valuable customizable biomaterial for future applications in tendon repair.

## 1. Introduction

Tendons play a vital role in the ankle movement. Acute and chronic sports-related tendon injuries are becoming more frequent in people of all ages, often leading to repeated pain and even disability [1, 2]. Scar formation is common after a tendon injury, limiting biological performance [3]. At present, tendon injury treatment remains challenging for clinicians. Primary treatments include autologous and allogeneic tendon transplantation or artificial tendon replacement. However, these reconstructive techniques may cause loss of function at the donor site, infection, rejection, or poor graft integration [4]. Therefore, researchers have been developing new technologies for tendon regeneration in recent years. Tendon tissue engineering has emerged as a promising treatment modality [5].

Silk fibroin- (SF-) based biomaterials have been applied for tissue regeneration recently due to their excellent biocompatibility, controllable mechanical properties, and ease of processing [6–8]. SF biomaterials are available as films [9], sponges [10], and hydrogels [11]. The Corneal tissue [12, 13] and articular cartilage [3] have been reconstructed with SF film. The fiber structure of SF is similar to that of type I collagen [8], and the structure of SF film is similar to that of tendon sheaths, which play a crucial role in tendon regeneration [14]. The biological activity and physical properties of SF film are suitable for tendon regrowth [15, 16]; however, the effect of SF film microstructures on tendon regeneration has not been thoroughly evaluated.

Studies have shown that biomaterials with microstructures mimicking native structures would allow for early core cell adhesion and proper cell biological behavior for tendon regeneration [17–19]. The tendon tissue has parallel aligned collagen fibers where tenocytes reside in the narrow space between collagen fibers [20, 21]. Tendon stem/progenitor cells (TSPCs) are the precursor cells for tendon regeneration [22–24] and have been used as seed cells for tendon tissue engineering. In this study, we prepared SF films with different bionic microstructures and mechanical properties mimicking healthy rat tendons and then investigated their biological effects on rat TSPCs to explore potential applications in human tendon regeneration.

## 2. Materials and Methods

### 2.1. Animals

Animals were provided by the Animal Center of the Third Military Medical University. A total of 5 four-week-old male Sprague–Dawley (SD) rats were sacrificed to extract TSPCs. Additionally, 10 eight-week-old male SD rats weighing 200-250 g were sacrificed for scanning electron microscope (SEM) and tissue section staining. The Animal Research Ethics Committee of the Third Military Medical University approved all experimental procedures.

### 2.2. Isolation and Characterization of Rat TSPCs

A total of 5 male four-week-old SD rats were sacrificed to isolate TSPCs, as previously described [23]. Briefly, Achilles tendons from both hind feet were dissected after euthanasia. Only the mid-substance tendon tissue was harvested, and the peritendinous connective tissue was carefully removed. The harvested tissue was minced in sterile phosphate-buffered saline (PBS) and digested in 3 mg/mL of type I collagenase (Sigma-Aldrich, St. Louis, MO) for 2.5 hours at 37°C. A 70 mm cell strainer (Becton Dickinson, Franklin Lakes, NJ) was used to remove the undigested tissue. After three washes with PBS, the released cells were resuspended in Dulbecco's Modified Eagle Media (DMEM) (Gibco, Carlsbad, CA) supplemented with 10% fetal bovine serum (FBS), 100 U/mL penicillin, 100 mg/mL streptomycin, and 2 mmol/L L-glutamine (all from Invitrogen, Carlsbad, CA) and incubated at 37°C and 5% CO_2_ for 2 days. Nonadherent cells were removed using PBS. After 7 days, the cells were trypsinized with Trypsin-EDTA solution (Sigma-Aldrich) and used as passage 0 cells. Passages 3 (P3) cells were used for all subsequent experiments.

### 2.3. Trilineage Differentiation Assay

TSPCs were incubated with adipogenic, osteogenic, and chondrogenic induction medium as previously described to characterize their multilineage differentiation potential [25]. Briefly, TSPCs were seeded in six-well plates at a cell density of 2 × 10^4^ cells/cm^2^ before inducing differentiation. Then, the TSPCs were cultured in the appropriate induction medium and stained according to the respective adipogenic (RASMX-90031, Cyagen, Guangzhou, China), chondrogenic (RASMX-9004, Cyagen, Guangzhou, China), and osteogenic (RASTA-90021, Cyagen, Guangzhou, China) induction differentiation protocols. The TSPCs were then observed under a light microscope.

### 2.4. Fabrication of SF Film with Smooth or Bionic Microstructure Surfaces

Silk solution extraction and SF film microstructure fabrication were completed as previously described [26–29]. Briefly, protein extract from cocoons (supplied by State Key Laboratory of Silkworm Genome Biology, Southwest University) was cut into three segments and boiled in 0.02 M Na_2_CO_3_ (Aladdin Reagent Co. Shanghai, China) for 40 minutes. Next, the protein extract was rinsed in dH_2_O for 20 minutes and dried overnight at room temperature. The protein extract was then dissolved in 9.3 M lithium bromide (Aladdin Reagent Co. Shanghai, China) at room temperature and placed in a 50°C oven for five hours. Then, the solution was placed in cellulose dialysis membranes (Shanghai Tansoole Company, China) and dialyzed in water for 72 hours. Finally, the protein extract was centrifuged at 8000 r/min for 20 minutes to remove impurities. The resulting supernatant of aqueous silk solution had a final concentration of 4.5% wt./v. determined by gravimetric analysis and was stored at 4°C.

Silicon wafers with parallel ridge widths and spacing of 5 *μ*m, 10 *μ*m, 15 *μ*m, and 20 *μ*m, and 5 *μ*m groove depths (according to the data measured in step 2.3) were produced using standard photolithography techniques [27]. Polydimethylsiloxane (PDMS) molds were produced from these surfaces by casting 300 mL of a 10 : 1 mixture of potting to catalyst solution and then curing at 50°C for 4 hours. Smooth PDMS base plates and smooth silicon wafers were prepared as described previously [26–29].

PDMS plates with either smooth or microstructure surfaces were cut into 35 mm diameter casting surfaces, and 4 mL of silk solution was pipetted onto each surface. Postcasting, the SF films were water annealed for up to 100 minutes at 90°C as previously described [30, 31]. Afterward, the SF films measuring 100 *μ*m in thickness were removed from their respective PDMS molds and sterilized by UV irradiation for 2 hours before seeding with TSPCs.

### 2.5. Scanning Electron Microscopy (SEM)

Five eight-week-old male SD rats weighing 200-250 g were sacrificed for SEM. Rat Achilles tendons were isolated as described above. The specimens were fixed with 3% glutaraldehyde for 2 hours and rinsed twice with 0.1 M PBS for 15 minutes each. The specimens were then dehydrated (15 minutes each) in a series of ethanol solutions (50, 60, 70, 80, 90, 100%, and twice at 100%) and a series of tert-butanol solutions (50, 60, 70, 80, 90, 100%, and twice at 100%). The specimens were finally dried and placed on a sample stage. After drying, vacuum platinum plating was applied and observed with SEM (ZEISS-Crossbeam 304, ZEISS, Germany).

SF film samples were sputter-coated with gold for 60 seconds and observed under SEM (Phenom Prox, Phenom, Netherlands) at 15 kV. The thickness of the SF films was measured from their cross-sections, and the samples were tiled to observe their surface morphology. The thickness, width, and spacing of the SF film bionic microstructure were measured using ImageJ software.

### 2.6. Hematoxylin-Eosin (HE) Staining

Five eight-week-old male SD rats were sacrificed, and their Achilles tendons were harvested as described above. Tendon specimens were fixed in 10% formaldehyde for at least 24 hours at room temperature and dehydrated with an ascending alcohol gradient. Finally, the specimens were embedded in paraffin, which were cut into 3 *μ*m sections and then stained according to the manufacturer's protocol. All of the sections were examined using a light microscope (Olympus, Japan). Three fields on each section were randomly selected to measure the diameter of the collagen fibers using ImageJ software.

### 2.7. Mechanical Test

Mechanical testing of normal Achilles tendons and different SF films was performed as previously described [32]. In brief, Achilles tendons with bony attachments were isolated from five SD rats. The calcaneal and tibial ends of the tendons were fixed to two serrated jaws (Supplementary Figure 1), which were connected to the testing machine (*E1000*, Instron, USA). The serrated jaws could be adjusted using a grip to achieve stable fixation. Before testing, the SF films were water annealed at 90°C for 100 minutes as previously described to improve their mechanical strength [31]. The cut SF film specimens were rolled and gently pressed into flat strips with a similar length, width, and thickness as the natural Achilles tendon (1 cm in length, 2 mm in width, and 1 mm in thickness) and then secured to the serrated jaws. The testing machine was used to evaluate the tensile stress-strain curves for all specimens as previously described [17, 33].

### 2.8. Cell Viability Assay

TSPCs were cultured on tissue culture plastic (TCP), smooth SF films, and SF films with different microstructure surfaces (5 *μ*m, 10 *μ*m, 15 *μ*m, and 20 *μ*m) at a density of 1 × 10^4^ cells/cm^2^ for 1, 2, and 3 days. Cell viability was measured with a Cell Counting KIT-8 (CCK-8, Dojindo, Japan). Briefly, TSPC or SF film-TSPC constructs were harvested at the designated time points. After incubation with 10% CCK-8 solution at 37°C for 2 hours, 100 *μ*L of the solution was transferred to a new 96-well plate to measure the absorbance at 450 nm using a microplate reader (Model 680, Bio-Rad, USA).

### 2.9. Immunofluorescence of TSPCs and Measurement of Cell Morphology

To characterize the surface marker expression of the TSPCs, the specific expression levels of CD34 (Anti-CD34 antibody, 1 : 200, ab81289, Abcam, Cambridge, UK), CD44 (Anti-CD44 antibody, 1 : 200,ab216647, Abcam, Cambridge, UK), CD3 (Anti-CD3 antibody, 1 : 200, ab135372, Abcam, Cambridge, UK), and CD90 (Anti-CD90/Thy1 antibody, 1 : 200, ab225, Abcam, Cambridge, UK) were detected by immunostaining. Cells were fixed with 4% paraformaldehyde (PFA), permeabilized with 0.1% Triton­X and incubated with primary antibody (1 : 1000). An Alexa Fluor® 488-conjugated goat anti­rabbit IgG (ab150077) secondary antibody was used at a dilution of 1 : 1000. Stained cells were observed under an inverted fluorescence microscope.

TSPC staining was completed as previously described [25]. Briefly, after adhering to TCP or the SF films for 24 hours, cells were fixed with 4% PFA for 20 minutes at room temperature and then permeabilized with 0.5% Triton X-100 for 5 minutes. Then, the cells were incubated in 100 nM rhodamine phalloidin (Yeasen Biological Technology Co, Shanghai China) for 30 minutes to stain the actin cytoskeleton. Nuclei were counterstained with 100 nM DAPI (Beyotime Biotech, Jiangsu, China) for 5 minutes. Images were obtained with a laser scanning confocal microscope (Zeiss lsm780, Germany) and analyzed with ImageJ software to measure the cell body aspect ratios (length/width), cell body major axis angles, and cell area [17, 24]. All measurements were obtained from 20 cells per image, and three images were analyzed from each group.

### 2.10. Real-Time Quantitative Polymerase Chain Reaction (RT-qPCR)

The mRNA expression levels of tendon-related genes of collagen type I alpha china (COL1A1), tenascin-C (TNC), tenomodulin (TNMD), and scleraxis (SCX) were determined using real-time quantitative polymerase chain reaction (RT-qPCR). One microgram of total RNA was extracted from TSPCs using TRIzol reagent (TaKaRa, Dalian, China) according to the manufacturer's protocol, and then 1 *μ*g of RNA was converted to complementary DNA (cDNA) using a Superscript III First-Strand Synthesis Kit (TaKaRa). qPCR was performed using a SYBR Green RT-PCR kit (TaKaRa) and an ABI Prism 7900 Sequence Detection System (PE Applied Biosystems, Foster City, CA, USA). The housekeeping gene glyceraldehyde 3-phosphate dehydrogenase (GAPDH) was used as an internal control to calculate the relative expression level of the target gene. The PCR primer sequences are shown in Table 1.

### 2.11. Statistical Analysis

Unless stated otherwise, all experiments were performed in triplicate, and the data were presented as the mean ± standard deviation. Quantitative data were analyzed using analysis of variance (ANOVA) with SPSS 22.0. A *p* value less than 0.05 was considered statistically significant.

## 3. Results

### 3.1. Rat TSPC Multilineage Differentiation Potentials

**Figure 1:**
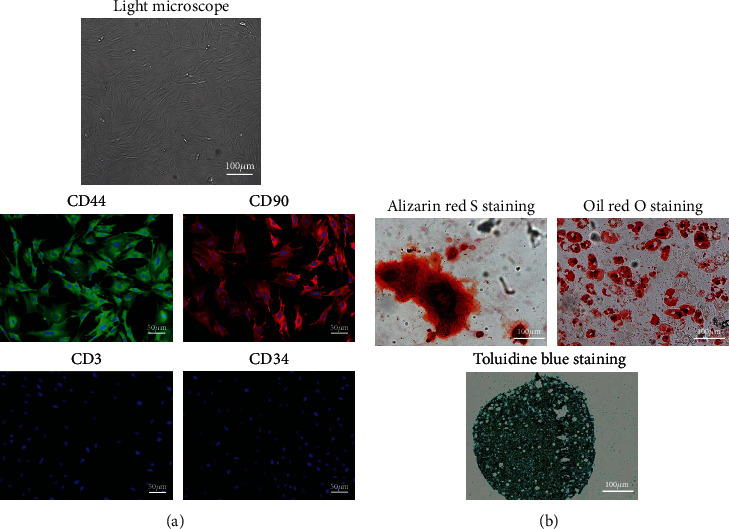
Cell morphology of tendon stem and progenitor cells on tissue culture plates under light microscope and immunofluorescence staining of CD44, CD90, CD3, and CD34 markers (a). Alizarin red S, oil red O staining, and toluidine blue staining after induction of osteogenesis, adipogenesis, and chondrogenesis (b).

At lower density, the TSPCs exhibited fibroblast-like spindle shapes. At 80% to 90% confluence, the TSPCs exhibited a pebble-like morphology and developed tight colonies. Immunostaining of specific surface antigens (CD44, CD90, CD3, and CD34) was used to characterize the newly isolated rat TSPCs. The TSPCs were positive for CD44 and CD90, but negative for the hematopoietic stem cell marker CD34 and the leukocyte marker CD3 (Figure 1(a)).

TSPCs were incubated in specific lineage induction medium for 14 days to characterize their multilineage differentiation potentials (Figure 1(b)). The TSPCs were positive for alizarin red S staining, indicating calcium deposition. The TSPCs also displayed round orange cytoplasmic droplets upon oil red O staining, suggesting lipid droplet formation. Additionally, blue-stained acidic glycosaminoglycans were observed, consistent with extracellular matrix formation during chondrogenesis.

### 3.2. SEM, HE Staining, and Biomechanical Testing of Rat Achilles Tendons

**Figure 2:**
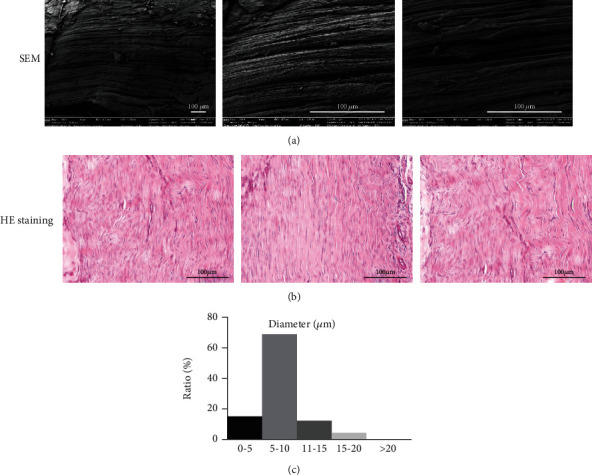
Fibrous structure of native Achilles tendon evaluated with scanning electron microscope (SEM) (a) (magnification at ×100, ×500, and ×500) and HE staining (b) (magnification at ×200). The fiber diameter distribution of native tendons was measured (c).

SEM was used to examine the morphology of healthy rat Achilles tendons. Collagen fibers in native rat Achilles tendons were arranged tightly in parallel with an even thickness (Figure 2(a)). A few visualized wavy collagen fiber bundles may have been secondary to a relaxed state.

Native rat Achilles tendon tissue sections were stained with HE staining. The normal tendon structure demonstrated ordered arrangement of the collagen fibers (Figure 2(b)). Collagen fiber diameters ranged from 5 to 20 *μ*m, and about 70% of the fibers had a diameter of 5-10 *μ*m (Figure 2(c)).

### 3.3. Characterization of SF Films with Different Microstructures Using SEM and Biomechanical Tests

**Figure 3:**
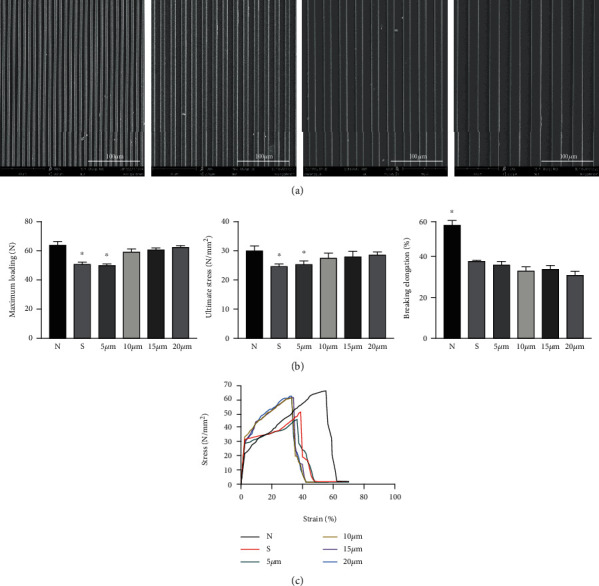
SEM images of SF films with bionic microstructures at 5, 10, 15, and 20 *μ*m, respectively (magnification at ×1000) (a). The maximum loading, ultimate stress (N/mm^2^), and breaking elongation (%) of SF film groups and native tendon were measured (b). The strain-stress curves of samples in different groups (c). Group N was the native tendon group, group S was the SF film group with a smooth surface, and the other groups were SF film groups with different microstructure sizes (5, 10, 15, and 20 *μ*m). ^∗^ indicates *p* < 0.05.

SF film morphology was characterized with SEM (Figure 3(a)). SF films successfully replicated the features defined on the PDMS substrates as the microstructure pitch of the SF films ranged from 5 to 20 *μ*m. In addition, there was ordered arrangement of the bionic structures on the SF film surface.

To compare the mechanical properties between native tendon and SF films, we performed mechanical tests including maximum loading, ultimate stress (N/mm^2^), and breaking elongation (%) on native tendon (N), smooth SF film (S), and SF films with different microstructure diameters (5 *μ*m, 10 *μ*m, 15 *μ*m, and 20 *μ*m). SF films with microstructure diameters (10 *μ*m, 15 *μ*m, and 20 *μ*m) exhibited comparable maximum loading and ultimate stress as the native tendon, with the exception of the smooth and 5 *μ*m SF film groups. As the width of the bionic groove increased, mechanical properties such as the maximum loading capacity increased gradually. However, the native tendon group had a significantly higher breaking elongation rate than the other groups (Figures 3(b) and 3(c)).

### 3.4. Cell Viability and Morphology of TSPCs on SF Films

**Figure 4:**
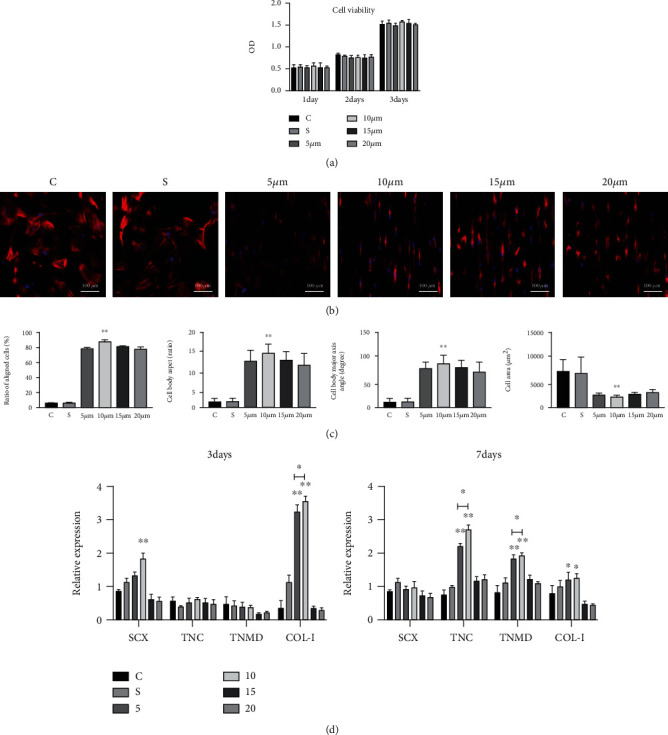
Cell viability on days 1, 2, and 3 was detected using the CCK-8 assay in different groups (a). Cytoskeleton and nucleus staining of TSPCs on different SF film groups and control group and also the distribution of normal tendon fibers and tendon cells (b). Quantitative analysis of the ratio of oriented aligned cells, cell body aspect, major axis angle, and cell area of TSPCs in different groups (c). Comparison of the expression levels of tendon-related genes after 3 days and 7 days (d). Group C was the normal culture plate group, group S was the SF film group with a smooth surface, and the other groups were SF film groups with different microstructure sizes (5, 10, 15 and 20 *μ*m). ^∗^ indicates *p* < 0.05, ^∗∗^ indicates *p* < 0.01.

CCK-8 was used to assess the cell viability of TSPCs grown on SF film at different time points. TSPCs adhered well to the surface of SF films with different microstructures and proliferated with time. No significant difference in cell viability was observed between the experimental groups and the control group at each time point (Figure 4(a)).

We performed immunostaining of cytoskeletal proteins F-actin and counterstained with DAPI for nuclei to further characterize the morphology of TSPCs on different SF films. The TSPCs exhibited a polygonal shape when grown on the surface of smooth SF films and on ordinary cell culture plates, but demonstrated an elongated cell morphology on SF films with different microstructure surfaces. The TSPCs exhibited a similar cell arrangement and morphology as in normal tendons, especially in the 10 *μ*m SF film group (Figure 4(b)).

We further quantified the morphological changes in TSPCs on different SF film surfaces using the ratio of aligned cells (%), cell body aspect ratios (length/width), cell body major axis angle (degree), and total cell area (*μ*m^2^) (Figure 4(c)). Compared with the control group and the smooth SF film group, TSPCs grown on the bionic microstructure SF film demonstrated an oriented arrangement and slender cell morphology. Among the four different microstructure sizes, cells grown on the 10 *μ*m microstructure surface had the highest ratio of aligned cells, cell body aspect ratio, cell body major axis angle, and the smallest cell area (*p* < 0.01 for all). These data suggest that SF films with a bionic microstructure can alter cell orientation and morphology. The TSPCs had the best biologic effects on the 10 *μ*m microstructure SF films.

We also evaluated the tendon-related gene expression of TSPCs in different groups using qRT-PCR. At 3 days, the early expression of the tendongenesis marker *SCX* was significantly higher than that in the control and other SF films groups, and *COL1A1* was also significantly higher in the 5 *μ*m and 10 *μ*m groups (*p* < 0.01). At 7 days, other than *COL1A1*, the expression levels of tendon-specific markers *TNC* and *TNMD* were also significantly higher in the 5 *μ*m and 10 *μ*m groups. The 10 *μ*m group had the highest expression among all the groups (*p* < 0.01) (Figure 4(d)).

## 4. Discussion

Although various biomaterials have been evaluated for tendon regeneration, the regenerated SF film is the most promising thus far [15, 29, 34–36]. In this study, we first isolated and characterized TSPCs from the native tendon of SD rats. We also evaluated the structure and mechanical properties of native tendon using SEM and HE staining. We then prepared SF films with different bionic microstructure sizes based on the parameters of the native tendon and evaluated the cell viability, cell morphology, and tendon marker gene expression of rat TSPCs. Our results demonstrate that SF film can mimic the structure of native tendon and has no cell toxicity. The 10 *μ*m SF film group had the highest percentage of oriented TSPCs and the most significant effect on cell morphology and also induced the highest expression of tendongenesis markers.

Previous studies have investigated SF for tendon repair, mostly by mixing it with other materials (such as PLA) and using electrospinning technology to prepare electrodischarge fibers [37–39]. However, the degradation and biocompatibility of mixed materials are not as good as those of pure SF, and the impact on the biological behavior of cells is volatile. We prepared pure SF films and accurately prepared microstructures on the surface to better understand the influence of SF and its microstructure on cells.

Biomaterials play a pivotal role in providing a mechanical framework for promoting soft tissue healing [35, 40, 41]. Thus, SF films should have similar mechanical properties as the native tendon tissue to promote tendon regeneration. However, typical SF films have high water solubility and low mechanical properties due to their *α*-helix predominance, which is not optimal for tendon regeneration. According to previous studies [27, 30, 31], the *β*-sheet content can be increased through water annealing treatment, improving the mechanical properties of SF film. In this study, water annealing treatment was applied at 95°C for 100 minutes. The final mechanical properties of SF films with a thickness of 100 *μ*m were comparable to those of native rat Achilles tendon. SF films provide mechanical support to reduce minor secondary damage caused by local instability and can be used for tendon regeneration [40, 42].

Previous studies have used bone marrow-derived stem cells (BMSCs) [35, 43, 44] as seed cells, which play a crucial role in tissue engineering [45, 46]. In this study, TSPCs, which are stem and progenitor cells within the tendon tissue [23], were used as seed cells to evaluate the biological effects of SF film on tendon regeneration. Compared to BMSCs, TSPCs have a higher tendon differentiation potential and are the primary functional cells in tendon reconstruction [22, 23]. Thus, TSPCs may simulate cell-material interactions in vivo and can be used to assess the biological performance of SF film in tendon regeneration more accurately.

In previous studies, growth factors and chemical groups were added to biomaterials to regulate the biological behavior of cells, but they were easily inactivated and the effects were unstable [47, 48]. In this study, microstructures were constructed on the SF film surface to provide physical stimulation signals for the TSPCs, and the resulting biological effect was more stable and controllable. Biomaterials interact with seed cells, causing morphological changes and changes in the cell function [29, 49, 50]. Our study indicates that the bionic microstructure of SF films can affect cell morphology and arrangement. As previously demonstrated, elongated cell morphology and oriented cell arrangement are conducive to the tendon differentiation of TSPCs and ordered deposition of the extracellular matrix [17, 44]. As such, the use of SF films for tendon regeneration may be more effective with the construction of bionic microstructures according to the native tendon fiber sizes. By interacting with bionic microstructures on the SF films, TSPCs exhibited directional alignment and a narrow cell morphology similar to normal tendon cells and unlike the behavior of the control and smooth SF film groups.

Not surprisingly, the results of qRT-PCR also corroborated our cell morphology observations. The 10 *μ*m SF film group promoted the expression of early tendon differentiation markers including transcription regulators scleraxis and type I collagen, while the expression levels of tenescin-C and tenomodulin increased in both the 5 *μ*m and 10 *μ*m SF films groups at a later stage of differentiation [25]. Based on morphological analysis, we postulated that the varying effects on differentiation regulation were mainly due to changes in cell morphology. The 10 *μ*m SF films had the most dramatic effect on the spatial arrangement and morphology of TSPCs.

SF films have good biological activity and mechanical properties. The presence of bionic microstructures on their surfaces enabled them to provide significant biological guidance for TSPCs and to serve as a potential material for tendon repair. However, this study was limited to rat tendons. Future studies should focus on optimizing the bionic microstructure size for repairing human tendon injuries.

## 5. Conclusions

Our study confirmed the feasibility of mimicking the properties of the native tendon tissue through the fabrication of SF films with bionic microstructures, providing a promising biomaterial for tendon tissue engineering and regeneration.

## Figures and Tables

**Table 1:** Primers used in qPCR analysis.

Gene name		Annealing temperature (°C)	PCR product size (bp)
*TNMD*	F:TGTACTGGATCAATCCCACTCT R:GCTCATTCGGGTCAATCCCCT	60	115
*SCN*	F:CCTTCTGCCTCAGCAACCAG R:GGTAGTGGGGCTCTCCGTGACT	60	156
*COL1A1*	F:GGCGGCCAGGGCTCCGACCC R:AATTCCTGGTCTGGGGCACC	60	320
*TNC*	F:CAAGGGAGACAAGGAGAGTG R:AGGCTGTAGTTGAGGCGG	60	159
*GAPDH*	F:GACTTCAACAGCAACTCCCAC R:TCCACCACCCTGTTGCTGTA	60	125

## Data Availability

The datasets generated and analyzed during the present study are available from the corresponding author upon reasonable request.

## Supplementary Materials

Supplementary Materials Supplementary Figure 1: serrated jaw of the testing machine: the serrated jaw was connected to the end of sample; the grip was adjusted a to achieve stable fixation.
