# Supplementary figures and images for: Bionic Silk Fibroin Film Induces Morphological Changes and Differentiation of Tendon Stem/Progenitor Cells
Source: Appl Bionics Biomech. 2020 Dec 1;2020:8865841. doi: 10.1155/2020/8865841 (PMC7725557; doi:10.1155/2020/8865841)

serrated jaws

grip

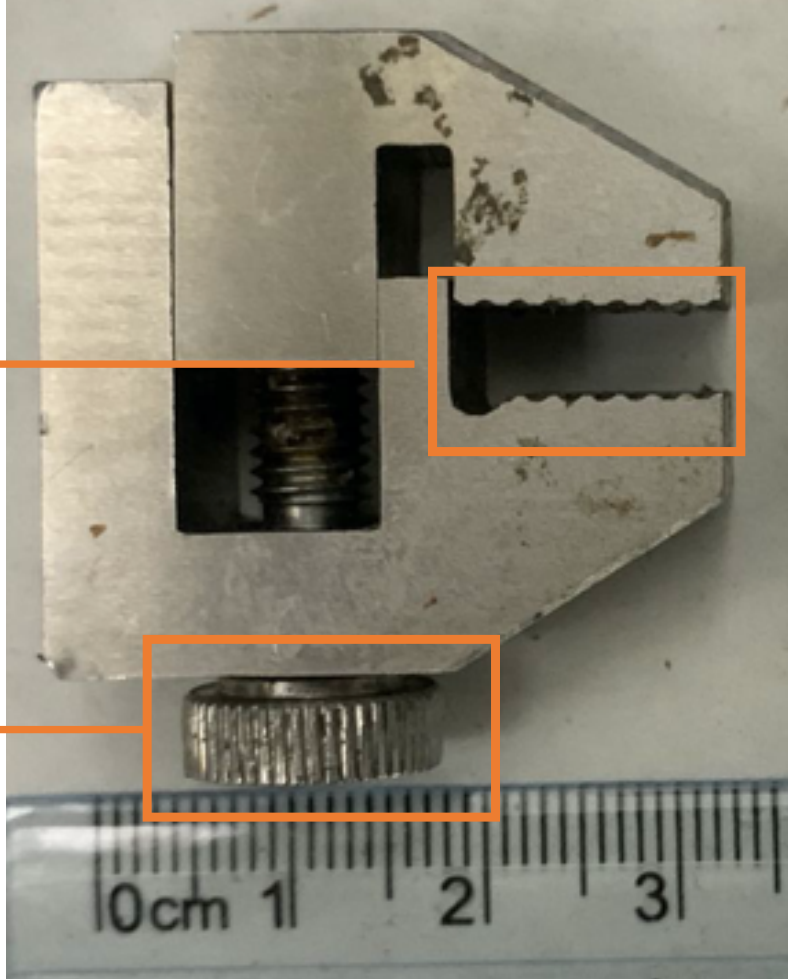

Supplement: Supplementary Materials — Supplementary Figure 1: serrated jaw of the testing machine: the serrated jaw was connected to the end of sample; the grip was adjusted a to achieve stable fixation. [file 8865841.f1.pdf]
